# Under Disguise: A Concealed Case of Acute Hepatitis A Infection

**DOI:** 10.7759/cureus.48224

**Published:** 2023-11-03

**Authors:** Jordan C Malone, Ernesto Zamora, Joseph M Gosnell, Heather L Stevenson, Akshata Moghe

**Affiliations:** 1 Department of Internal Medicine, University of Texas Medical Branch at Galveston, Galveston, USA; 2 Department of Gastroenterology and Hepatology, University of Texas Medical Branch at Galveston, Galveston, USA; 3 Department of Pathology, University of Texas Medical Branch at Galveston, Galveston, USA; 4 Department of Gastroenterology and Hepatology, University of Texas Health Science Center at Houston, Houston, USA

**Keywords:** anchoring bias, hepatology, hyperbilirubinemia, acute cholecystitis, hav

## Abstract

Acute hepatitis A virus infection is routinely identified through a thorough patient history in conjunction with liver chemistries and viral serologies. The diagnosis has the potential to be delayed when the clinical picture is obscured with another, seemingly more urgent presenting pathology with overlapping features. Here, we describe the case of a young female who presented with acute calculous cholecystitis with concurrent acute hepatitis A virus infection.

## Introduction

Acute hepatitis A virus (HAV) infection is a well-known, vaccine-preventable viral infection stemming from fecal-oral transmission. Since 2016, HAV outbreaks have been publicly reported in 37 states, totaling 44,915 cases, of which 61% required hospitalization and 423 led to death [1]. The natural disease course of HAV includes an incubation period that can last up to one month, followed by a prodromal phase of nonspecific flu-like symptoms with gastrointestinal involvement, followed by an icteric phase with hepatic injury as evidenced by elevated liver chemistries and jaundice [2,3]. The diagnosis of acute HAV infection is typically elicited by a careful history and examination, with routine and serological blood work. However, the presence of a concomitant gastrointestinal condition can confound the picture resulting in a delay in diagnosis and management. Here, we present a case of acute HAV presenting concurrently with acute cholecystitis.

## Case presentation

**Figure 1:**
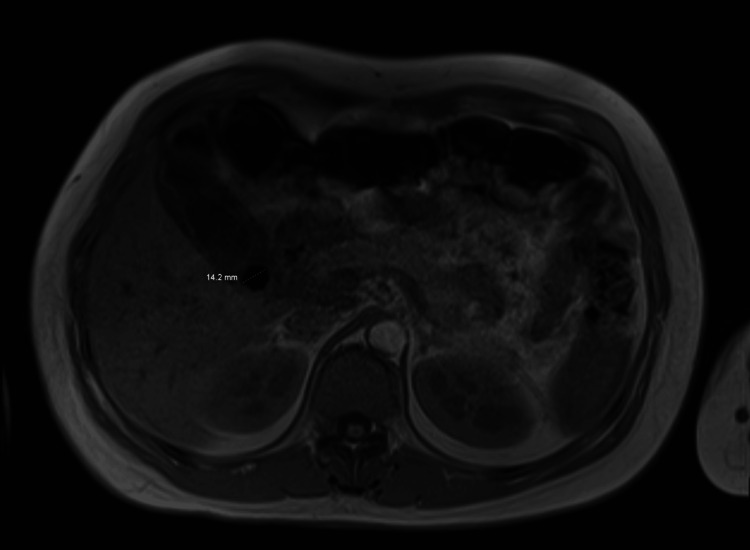
MRI abdomen Magnetic resonance imaging of the abdomen with and without contrast, demonstrating a 14.2 mm stone in the gallbladder neck

A 36-year-old female with no significant past medical history presented with jaundice and mild, sporadic epigastric abdominal pain of three-day duration with associated dark urine and pale-colored stools. Physical examination revealed an afebrile, hemodynamically stable woman with point tenderness in the right upper abdominal quadrant. Workup was significant for a total bilirubin of 13.5 mg/dL (direct 9 mg/dL), alkaline phosphatase of 169 U/L, gamma-glutamyl transferase (GGT) of 136 U/L, alanine transaminase (ALT) of 586 U/L, and AST of 100 U/L. Ultrasound of the liver and gallbladder, as well as magnetic resonance cholangiopancreatography (MRCP), were significant for cholelithiasis with a 14.2 mm (1.42 cm) echogenic stone identified at the gallbladder neck with changes of acute cholecystitis (Figure 1).

No biliary ductal dilation was noted on the MRCP, and hence endoscopic retrograde cholangiopancreatography (ERCP) was not deemed necessary. The patient was taken directly for laparoscopic cholecystectomy with intraoperative cholangiography and liver biopsy on hospital day two. Intraoperative findings were consistent with acute cholecystitis without filling defects in the biliary tree.

**Figure 2:**
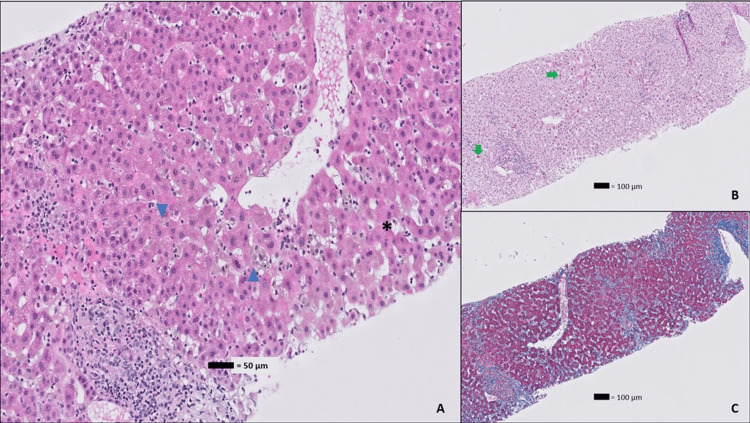
Hepatic histopathology Image A: Portal tract and lobule with dilated sinusoids and several foci of severe hepatocanalicular cholestasis (blue triangles) and apoptotic hepatocytes (black asterisk) (H&E stain, 20X magnification); Image B: Portal tract and lobule with numerous ceroid-laden macrophages (green arrows) (PAS-D stain, 10X magnification); Image C: Trichrome stain demonstrating centrivenular fibrosis and lobular edema (trichrome stain, 10X magnification). Histological findings are consistent with a diagnosis of active hepatitis A. H&E: hematoxylin and eosin PAS-D: Periodic acid-Schiff with diastase

**Figure 3:**
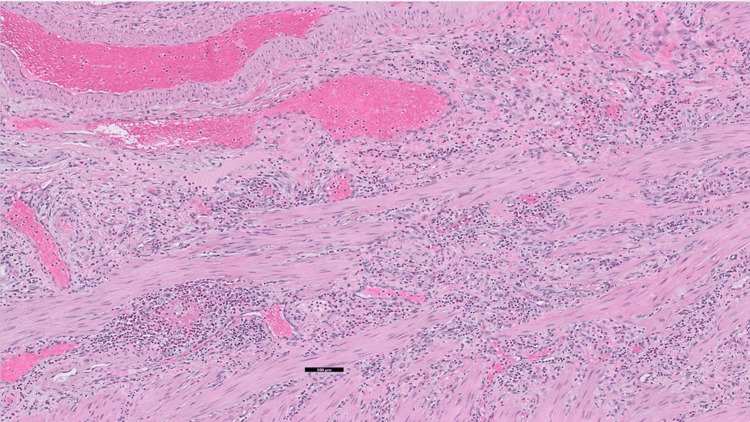
Gallbladder histopathology Photomicrograph of abundant eosinophils infiltrating the gallbladder wall muscular layer (H&E stain, 40X magnification)

The patient’s bilirubin continued to rise post-cholecystectomy, peaking at 21.8 mg/dL. The hepatology team was then consulted and a viral hepatitis work-up was performed which returned positive for HAV immunoglobulin M (IgM). At the same time, her intra-operative liver biopsy also returned demonstrating acute viral hepatitis and frequent eosinophils in the muscular wall of the gallbladder (Figures 2, 3).

A diagnosis of acute hepatitis A infection was confirmed, and the patient was treated symptomatically. Her AST and ALT gradually trended down over the subsequent few days. Her symptoms resolved, and she was discharged with plans for close clinic follow-up. Further history revealed that she had consumed frozen strawberries from a local grocery store, and recent news had implicated the spread of HAV via frozen strawberries from certain farms in the area.

## Discussion

Despite lab work suggestive of hepatocellular injury and severe hyperbilirubinemia with jaundice, the diagnosis of HAV was delayed due to the concurrent presentation of acute cholecystitis and lack of a prodromal phase typical of HAV infection. Our case highlights the fallacy of an “anchoring bias,” wherein concurrent conditions are not considered because of complete reliance on the first piece of information received [4]. Although the imaging diagnosis of cholecystitis was noted first, it is important to consider other possible diagnoses when the history and lab work do not completely align with the clinical picture. Severe hyperbilirubinemia, as in this case, warrants further investigation as a bilirubin level greater than 2 mg/dL is not typical of acute cholecystitis [5]. Further workup with MRCP revealed no biliary filling defects in this case, therefore effectively ruling out choledocholithiasis. Histopathology of the gallbladder specimen, in this case, showed frequent eosinophils invading the muscular layer of the gallbladder. A rarer diagnosis of eosinophilic cholecystitis can be made via histopathological analysis when more than 90% of the cellular infiltrate consists of eosinophils, but this is generally in the absence of gallstones [6]. Our case may represent a localized eosinophilic reaction to the causative stone within the gallbladder neck. Because of anchoring bias, hepatitis serologies were not evaluated until two days following admission. Though our patient was eventually accurately diagnosed and discharged without complication, missed or delayed diagnoses can be costly in patients who are elderly, immunocompromised, or with preexisting liver disease. Although there was a significant decrease in the incidence of acute HAV infection by 43% from 2020 to 2021, acute HAV cases remain four times higher in the United States in 2021 as compared to 2015 [7].

## Conclusions

This case serves to illustrate the importance of further investigation of viral hepatitides in the setting of severe hyperbilirubinemia and jaundice, even if another related acute pathology is identified. In this particular case, the concurrent presentation of acute cholecystitis delayed the diagnosis of acute HAV infection likely secondary to symptom and laboratory result overlap as well as the urgency of the former diagnosis. A delay in proper diagnosis can be costly for patients who are elderly, immunocompromised, or those with pre-existing liver disease, therefore reiterating the importance of a timely and accurate diagnosis.

This case also demonstrates the pitfalls of an anchoring bias and should alert the clinician to retain a high suspicion for further investigation if the clinical workup remains inconsistent with a previously diagnosed process. The degree of hyperbilirubinemia demonstrated in this case is not consistent with acute cholecystitis and in the absence of common bile duct blockage, should prompt the clinician to search for alternative explanations that may better explain the clinical scenario. Despite the absence of typical features of acute viral hepatitis, all cases with this degree of hyperbilirubinemia should undergo serological testing for acute viral hepatitides.
